# How accurate is the phenotype? – An analysis of developmental noise in a cotton aphid clone

**DOI:** 10.1186/1471-213X-8-19

**Published:** 2008-02-23

**Authors:** Gregory A Babbitt

**Affiliations:** 1 Center for Evolutionary Functional Genomics, The Biodesign Institute, Arizona State University Tempe, AZ, 85287-5301, USA

## Abstract

**Background:**

The accuracy by which phenotype can be reproduced by genotype potentially is important in determining the stability, environmental sensitivity, and evolvability of morphology and other phenotypic traits. Because two sides of an individual represent independent development of the phenotype under identical genetic and environmental conditions, average body asymmetry (or "fluctuating asymmetry") can estimate the developmental instability of the population. The component of developmental instability not explained by intrapopulational differences in gene or environment (or their interaction) can be further defined as internal developmental noise. Surprisingly, developmental noise remains largely unexplored despite its potential influence on our interpretations of developmental stability, canalization, and evolvability. Proponents of fluctuating asymmetry as a bioindicator of environmental or genetic stress, often make the assumption that developmental noise is minimal and, therefore, that phenotype can respond sensitively to the environment. However, biologists still have not measured whether developmental noise actually comprises a significant fraction of the overall environmental response of fluctuating asymmetry observed within a population.

**Results:**

In a morphometric study designed to partition developmental noise from fluctuating asymmetry in the wing morphology of a monoclonal culture of cotton aphid, *Aphis gossipyii*, it was discovered that fluctuating asymmetry in the aphid wing was nearly four times higher than in other insect species. Also, developmental noise comprised a surprisingly large fraction (≈ 50%) of the overall response of fluctuating asymmetry to a controlled graded temperature environment. Fluctuating asymmetry also correlated negatively with temperature, indicating that environmentally-stimulated changes in developmental instability are mediated mostly by changes in the development time of individuals.

**Conclusion:**

The amount of developmental noise revealed in this trait potentially does interfere with a substantial amount of the sensitivity of fluctuating asymmetry to change in temperature. Assuming that some genetic-based variation in individual buffering of developmental instability exists in natural aphid populations, the amount of internal developmental noise determined in this study could also substantially reduce evolvability of the aphid wing. The overall findings here suggest that individual response to the seemingly high cost of stabilizing some aspects of the phenotype may account for the frequent observation of trait and species specificity in levels of fluctuating asymmetry.

## Background

### Developmental instability

Phenotype is determined partly by the interaction of genotype and environment and partly by random internal noise during development. The phenotype is also generally robust to the combined effects of mutation, environmental change, and internal noise [1]. This robustness is determined by the interplay of canalization and developmental stability, two types of developmental buffering that probably share underlying regulatory mechanisms but are functionally distinct [2], and also phenotypic plasticity, an adaptive change of phenotype in response to different environments [3]. Debat and David [4] define developmental stability as "a set of mechanisms historically selected to keep the phenotype constant in spite of small, random developmental irregularities potentially inducing slight differences among homologous parts within individuals." While the assumption that developmental stability is largely the result of selection may be debated, instability during development generally is thought to indicate stress. Primary interest in the subject of developmental instability has been fueled by its potential utility as a general environmental bioindicator of environmental or genetic stress [5-7] or as an indicator of good genes (i.e. stable development) within the context of mate choice [8,9]. Traditionally, the term "developmental instability" has been equated loosely with "developmental noise" [10], however, because developmental instability is often responsive to the environment and, yet, always exists to some degree in the absence of environmental changes, it must have both internally- and externally-driven components. In this paper, I restrict the definition of developmental noise to the internal component of developmental instability and investigate the internal accuracy of the developmental process in a relatively unstable morphological trait, the aphid wing.

Developmental instability most often is estimated by fluctuating asymmetry (FA), the right and left side difference in size or shape in a single trait across the population [6,11-13]. Generally, it is assumed that FA is the result of both measurement error and some level of genetic-based buffering of environmentally-linked noise during development. Therefore, the average level and variability of FA observed in any population potentially depends on four influences: 1) some normally distributed measurement error, 2) an environmental sensitivity in the development of the phenotype, 3) a genetically based and, presumably, variable capacity to buffer this sensitivity, and 4) some level of stochastic internal noise that always is potentially present during the developmental process, even in the absence of gene by environment interaction. In more modern studies of FA, measurement error is controlled carefully. However most estimates of FA reported in the literature cannot speak to the relative contribution of the remaining factors to overall FA within a sample because they usually are collected from genetically heterogeneous populations.

**Figure 1:**
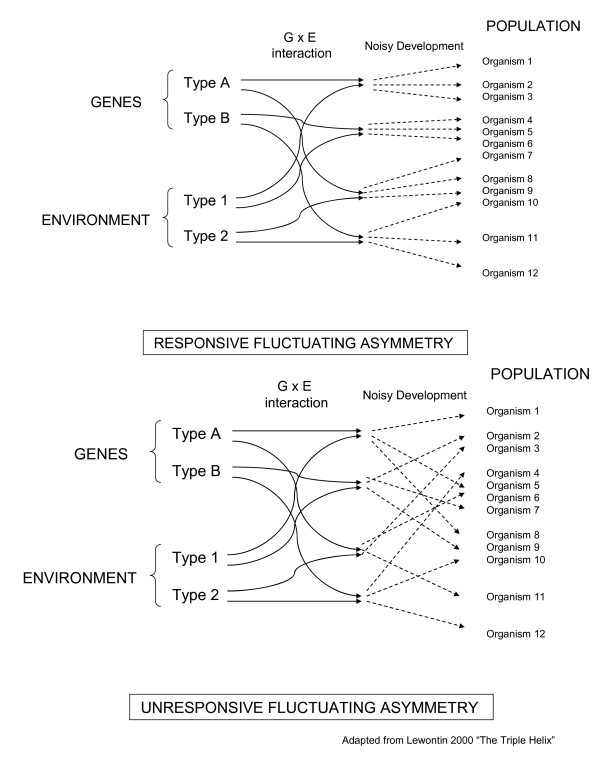
**The potential role of developmental noise in two models of fluctuating asymmetry within a population**. The environmental sensitivity and evolvability of fluctuating asymmetry both depend upon the amount of stochastic developmental noise relative to amount of variation imparted by differences in gene by environment interaction.

Biologists have often assumed either that FA is driven mostly by uniform individual responses to a variety of stessors encountered by a natural population in a heterogeneous environment or that FA is driven mostly by variability among individual capacities to buffer against a relatively uniform level of stress presented by a homogenous environment. This difference of opinion as to whether FA is mostly environmentally-based or has a significant genetic component is debated [14,15], and often seems to rest upon whether an author's primary aims are in demonstrating the utility of FA as a bioindicator of environmental stress or investigating whether organisms can use FA as an indicator of stable (i.e. "good") genes during mate choice. Both views implicity assume that the level of FA is highly reflective of the combination of gene and environment that occurs with each individual in a population, thereby also assuming the development of most traits is relatively robust to internal noise (Figure 1) [16]. This assumption that FA is not influenced heavily by internal stochastic processes during development is necessary if one is to hypothesize that FA could act as any kind of indicator (i.e. either environmental or genetic quality). Furthermore, this assumption largely remains unexplored due to the simple fact that the "individual" phenotype develops only once. However, in the absence of genetic variation, developmental noise could hypothetically be estimated through the comparison of intra-individual to inter-individual variation that represent, respectively, the influences of the internal and external environments on phenotype. Therefore, the response of FA in isogenic or monoclonal populations reared in a graded environment could be used to quantify the level of developmental noise within phenotypic traits and compare it to the response of developmental instability to the environment. Only a few studies have observed FA in isogenic or clonal organisms [2,7,17-20], and none for purpose of quantifying the role of developmental noise in comparison to phenotypic response to the environment.

### The basis of developmental noise

The body of a typically sized adult human is estimated to contain between 10 and 100 trillion cells. Building this body, therefore, requires roughly 50 doublings of the initial cell population (by comparison, an insect still may require ≈ 45 cell doublings). Milan et al. [21] has demonstrated that cell cycle synchrony is maintained in the *Drosophila *imaginal wing disc over an average distance of only 2–10 cells (2–4 doublings) regardless of the stage of development. This large difference between adult body size and the comparatively small extent of synchronized cell behavior implies a large potential for the accumulation of errors caused by random differences in the timing of cell cycles within expanding cell populations during growth and development. This source of developmental noise is amplified strongly by expanding populations of growing cells during the exponential growth phase, causing the multiplicative accumulation of developmental error. Without mechanisms to regulate this kind of noise, the ascertainment of an accurate and symmetric phenotype would be nearly impossible. However, empirical observations of the symmetry of various morphologies demonstrates that most organisms actually exhibit low levels of developmental instability, as estimated by FA. Clearly, while the potential for error that leads to developmental noise must be regulated dynamically during the growth process (see [22]), it is also probably never eliminated completely because of the lack of cell cycle synchrony between neighboring clusters of cells.

Despite the likely contribution of multiplicative error to FA, it has traditionally been assumed, that developmental instability somehow originates at a subcellular molecular level and that these effects are independent and additive in their contribution to overall body asymmetry [[11,12] and [23] but also see [24-26]]. Simply because the difference in scale between the size of molecules and the size of cells is so large, it would be unlikely that additive and independent effects of molecular noise (in a traditional Brownian sense) would comprise an important source of variation in the functioning of growing cells. This is because accuracy in the "laws" that describe physical-chemical systems has a relative probable error of $\frac{1}{\sqrt{n}}$ with n equal to the number of molecules cooperating to bring about the "law" [27]. Hence, the traditional explanation, that FA results from additive effects of chance events at the molecular level that accumulate to produce a normal distribution of FA would seem to lack logical grounding. However, Leamy and Klingenberg [28] rationalize that molecular noise could scale to the level of tissue when developmentally important molecules exist in very small quantities (e.g., regulated transcript or protein), and therefore FA actually may represent a stochastic component of developmental gene expression. The temporal variation in cell cycle that this could cause is also implicated by the observation of high kurtosis (or, more specifically, in this case a non-normal distribution due to power law scaling in the tails) often observed in FA data [25,29]. Babbitt [30] has also linked increased scaling phenomena (and kurtosis) to increased genetic heterogeneity in a population.

### Partitioning developmental noise from fluctuating asymmetry

Despite the potential for individual differences in gene and environment to influence the developmental stability of the phenotype, almost nothing is known about the overall level of internal developmental noise in a typical phenotypic trait. More importantly, how does this level of noise compare to the response of fluctuating asymmetry observed when an organism's environment changes? This is the central question in this investigation, which reports the percentage of variation in FA due to noise compared to the environmental response of FA along a temperature gradient in a genetically homogeneous population of organisms with complex morphology.

In this study, both the noise component of FA and its response to environment (temperature response) in the cotton aphid, *Aphis gossipyii*, is characterized. This species can reproduce parthenogenetically (apomictic) and often produces wings that are easily measured using multiple landmarks. Cotton aphids demonstrate large visible variation in body size, wing size, and wing FA, even within monoclonal cultures and are one of the few insects that demonstrate quite visible wing asymmetry within many individuals. Cotton aphids are also phenotypically plastic in response to temperature, producing smaller lighter morphs at high temperatures and larger, darker morphs at low temperatures. This unique feature allows observation of two genetically homogeneous groups in which differences in gene expression exist (causing the two color/size morphs). Therefore, this model system can allow for partitioning of the effect of variation in gene expression from variation in genotype on developmental stability.

## Methods

In March 2003, a monoclonal population of Cotton Aphids (*Aphis gossipyii *Glover) was obtained from Dr. J.P. Michaud in Lake Alfred, Florida and was brought to the Department of Entomology and Nematology at the University of Florida. The culture was maintained on cotton seedlings (*Gossipium*) grown at different temperatures (12.5°C, 15°C, 17°C, 19°C, 22.5°C and 25°C with n = 677 total or about 100+ per treatment) under artificial grow lights (14L:10D cycle). Because of potential under-sampling caused by a non-normal distribution of FA (see [29]), a second monoclonal population collected from Gainesville, FL in June 2004 was reared similarly, but in much larger numbers at 12.5°C, 15°C, 17°C, 19°C, and 25°C (n = 1677 or about 300+ per treatment).

Development time for individual apterous cotton aphids (Lake Alfred clone) were determined on excised cotton leaf discs using the method of Kersting et al. [31]. Twenty randomly selected females were placed upon twenty leaf discs (5 cm diameter) per temperature treatment. Discs were set upon wet cotton wool in petri dishes and any first instar nymphs (usually 3–5) appearing in 24 hours were then left on the discs. Development time was taken as the average number of days taken to reach adult stage and compared across temperatures. Presence of shed exoskeleton was used to determine instar stages. Cotton was wetted daily and leaf discs were changed every 5 days. Humidity was maintained at 50 ± 5%.

In each temperature treatment, single clonal populations were allowed to increase on plants until crowded, in order to stimulate alate (winged individuals) production. Temperature treatments above 17°C produced small light colored and tended to feed on the undersides of leaves of cotton seedlings. Temperature treatments below 17°C produced larger, dark morphs that tended to feed on the stems of cotton seedlings. Alatae were collected using small brushes dipped in alcohol and were stored in 80% ethanol. Wings were dissected using fine insect mounting pins and dry mounted as pairs on microscope slides. Dr. Susan Halbert at the State of Florida Department of Plant Industry in Gainesville, FL performed species identification.

Specimens were dried in 85% ethanol, and pairs of wings were dissected (in ethanol) and air-dried to the glass slides while ethanol evaporated. Permount was used to attach cover slips. This technique prevented wings from floating up during mounting, which might slightly distort the landmark configuration. Dry mounts were digitally photographed. Six landmarks were identified as the two wing vein intersections and four termination points for the third subcostal.

Wing vein intersections were digitized using TPSDIG version 1.31 [32]. Specimens damaged at or near any landmarks were discarded. Fluctuating asymmetry (FA) was calculated using multivariate geometric morphometric landmark-based methods. All landmarks are shown in Figure 3B. FA (FA 1 in [12]) was calculated as absolute value of (R – L) where R and L are the centroid sizes of each wing (i.e., the sum of the distances of each landmark to their combined center of mass or centroid location). In addition, a multivariate shape-based measure of FA, known as the Procrustes distance, was calculated as the square root of the sum of all squared Euclidean distances between each left and right landmark after two-dimensional Procrustes fitting of the data [12,13,33,34]. This method removed any difference that was due to size alone. Centroid size calculation, Euclidean distance calculation, and Procrustes fitting were performed using Øyvind Hammer's Paleontological Statistics program PAST version 0.98 [35]. Percent measurement error (ME) was computed as (ME/average FA) × 100 where

*ME *= (|*FA*1 - *FA*2| + |*FA*2 - *FA*3| + |*FA*1 - *FA*3|)/3

in a smaller subset (200 wings, each measured 3 times = *FA*1, *FA*2 and *FA*3) of the total sample. All subsequent statistical analyses were performed using SPSS Base 8.0 statistical software [36]. Unsigned multivariate size and shape FA as well as the kurtosis of signed FA were then compared at various temperatures using one-way ANOVA. Directional asymmetry (DA) was assayed by means of a paired t-test comparing the centroid size of right and left wings as also the Euclidian distances between landmarks 2 and 3, the most variable wing character, between right and left wings.

Finally, the noise-to- response ratio was calculated where "noise" refers to variance in FA within the temperature classes (an "intra-individual" variance within the aphid clone), and "response" refers to the overall variance of FA between temperature treatments (an "inter-individual" variance within the aphid clones). The fraction of FA due to noise was calculated as the intra-individual variance of FA within each treatment divided by the total variance of FA among all individuals in the study.

## Results

**Figure 2:**
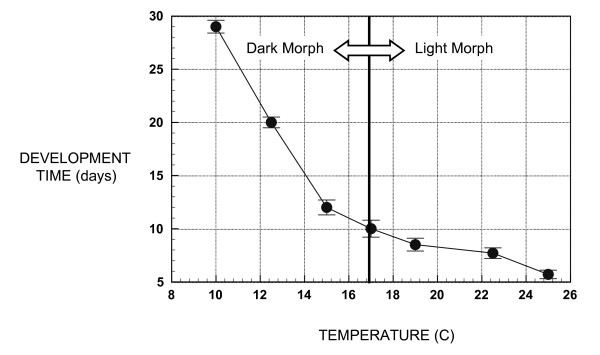
Cotton aphid mean development time ± 1 SE in days in relation to temperature (n = 531).

The overall fraction of FA due to developmental noise was 49.7% and 50.1% for size and shape FA, respectively, and the overall noise-to-response ratio was 1.00 and 1.02 for size and shape FA, respectively. There were no significant differences in the levels of developmental noise between light and dark morphs regardless of whether FA was measured using multivariate size or shape (Table 1). The development time curve (Figure 2) was very similar to previously published data [31,37], decreasing monotonically with temperature at a much steeper rate in dark morphs than in light morphs. Because dark morphs were, on average, 14.6% larger than light morphs (t = 18.522, p < 0.0001, df = 937) and because the relation of development time to temperature is more steeply sloped in dark morphs, it can be concluded that dark morphs achieve their greater size by increasing development time rather than by increasing their growth rate.

**Table 1:** The percentages of variance in fluctuating asymmetry due solely to internal developmental noise in wing size and shape in a cotton aphid clone. The percentage reported is the within "individual" variance in FA divided by the total variance in FA (= within "individual" + between "individual" variance).

**Temperature Celsius**	**Multivariate size**	**Multivariate shape**
12.5	54.8%	57.2%
15	53.2%	46.4%
17	50.1%	48.1%
19	44.7%	50.4%
25	46.4%	49.1%

**Total average across temperatures**	49.8%	50.2%

**Figure 3:**
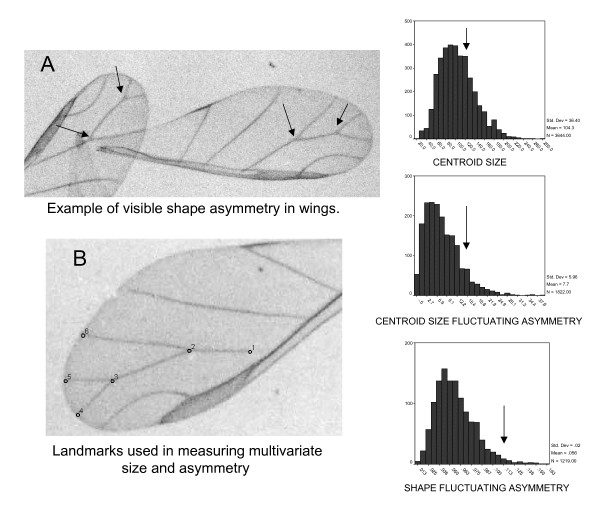
**(A) Example of wing asymmetry and (B) landmarks used in analyses of wings of cotton aphid *Aphis gossipyi***. Unsigned distribution of size, size-based and shape-based FA in monoclonal cotton aphids grown in controlled environment at different temperatures is also shown. Size and asymmetry values for the single pair of wings (pictured in A) is indicated by vertical arrows on the distributions to the right.

The shape of the distribution of centroid size, unsigned size FA, and shape FA appear similar exhibiting double Pareto lognormal distributions (Figure 3). See Babbit et al. [29], Babbit [30] and Reed and Jorgensen [38] for further explanation of this distribution type. Similar distributional patterns are observed within temperatures (not shown) as that observed across temperatures (Figure 3). Consistent with the prediction of Pertoldi et al. [20] regarding clonal populations, there is no significant correlation between size and FA in any temperature class. Scatter plots of (L+R) to (L-R) within each temperature class are all approximately circular, indicating approximately equal variances in size and FA as well (not shown).

**Figure 4:**
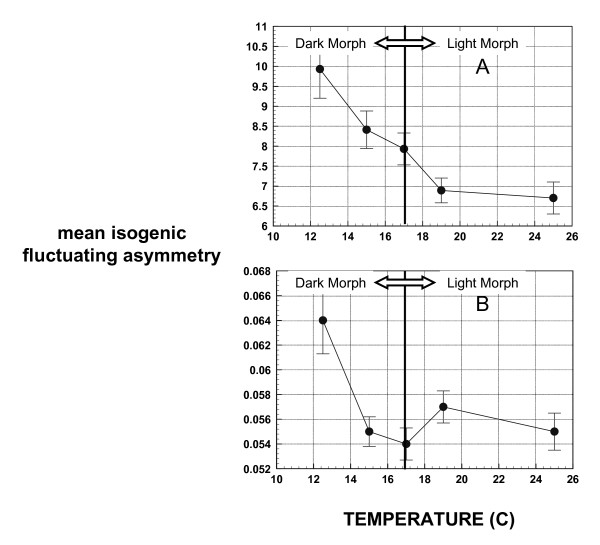
**(A) mean centroid size- based FA and (B) mean Procustes shape-based FA in monoclonal cotton aphids grown on isogenic cotton seedlings at different temperatures**.

Coefficient of variation for FA was slightly higher for dark morphs (12.5 C = 92.59%, 15 C = 93.02%, 17 C = 88.95%, 19 C = 79.02%, 22.5 C = 80.00% and 25 C = 85.35%), and mean isogenic FA (both size and shape) was highly significantly different across temperatures (ANOVA F = 6.691, df between group = 4, df within group = 1673, p < 0.001) in the Gainesville FL clone (Figure 4) but not in the Lake Alfred clone (ANOVA F = 1.992, df between group = 5, df within group = 672, p = 0.078). Because of probable under-sampling in the Lake Alfred clone (see [29]) the results presented here are from the Gainesville clone. Mean centroid size FA (Figure 4) and development time (Figure 2) follow a nearly identical pattern, decreasing rapidly at first then slowing with increased temperature.

**Figure 5:**
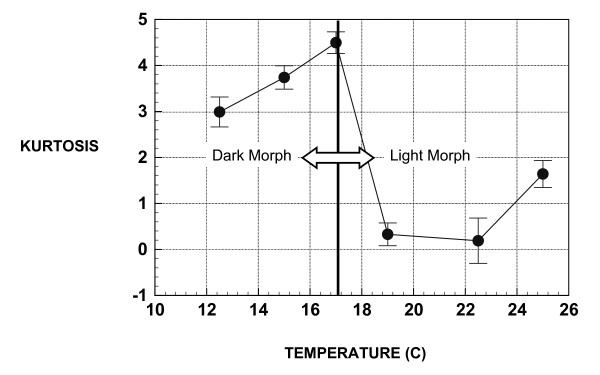
Kurtosis of signed distribution of size-based FA in monoclonal cotton aphids grown on isogenic cotton seedlings at different temperatures.

Mean shape FA was also significantly different across temperature classes (ANOVA F = 4.863, df within group = 4, df between group = 1673, p = 0.001) but this difference is due mostly to elevated FA in the 12.5°C group (Figure 4). Less than one percent of the variation in FA was due to variation in body size (r = -0.101 for shape FA; r = 0.088 for size FA). Kurtosis in the shape of the distribution of size based FA (Figure 5) was significantly higher in dark morphs than in light morphs (t = -2.21, p = 0.027). Within each morph (light or dark), kurtosis in the distribution of FA appears to increase slightly with temperature (Figure 5). Measurement error for shape FA was estimated at 2.2% and for size FA at 5.7%. There was no indication of directional asymmetry in the aphid wings.

## Discussion

### Developmental noise and the evolution of wing symmetry in aphids

Under the conditions of this experiment, the aphid wing was found to be a highly developmentally unstable trait, by comparison to levels of FA usually observed in the wings of other insects (see Figure 3), exhibiting nearly four times the level of FA that is typical of other insects [29]. This was clearly not the result of directional asymmetry possibly inflating the overall FA measured in this study. The surprisingly high noise-to-response ratios discovered here, combined with relatively low measurement error, also indicated that nearly half of the response of FA to environmental temperature could be also generated by internal stochastic developmental noise. Therefore, this level of developmental noise could impose a significant lack of sensitivity of FA, to either environment changes and/or genetic differences, in the ability to buffer development between clonal populations. If developmental noise varies across species and traits, this may explain why FA often has been observed to be highly species and trait specific [14]. High percentages of developmental noise in aphid wing symmetry are also interesting because they may imply that the high FA in the aphid wing may also be associated with a lack of evolvability on this trait, due to the interference that a noisy phenotype would have on any selection pressures imposed on the population. This may be rather unusual in the evolution of insect wings, as aphids are notoriously weak flyers and use their wings primarily for passive dispersal on wind currents. Therefore, there may be no real need for accurate development of wings in this group. This further suggests that, in general, stable development may come at some unknown physiological cost, perhaps a cost that some organisms, like aphids, refuse to pay when selection is relaxed. The observation of winged individuals in other aphid species that lack flight muscles and thus are incapable of powered flight [39] would also seem to support the hypothesis of relaxed selection on wing symmetry in aphids. This type of elevated response of wing FA to relaxed selection has also been observed in flightless beetles [40].

### Phenotypic plasticity and developmental noise

The comparison of developmental noise between the two phenotypically plastic color/size morphs in this species indicates no differences in developmental noise regarding wing size but a large difference regarding wing shape, with the development of a specific shape being much noisier in the light (high temperature) morphs. Temperature trends in mean FA, which are sloped differently in the two color/size morphs (Figure 4), were also found to be different regarding whether a size or shape-based approach was used. Additionally, while the low levels of kurtosis in the distribution of FA are consistent with those of a genetically homogeous population (see [30]), it is interesting that the average kurtosis differs significantly between the light and dark aphid morphotypes (Figure 5). In combination, all of these observed differences in the response of wing symmetry to temperature in these two isogenic, but differentially expressed genotypes may indicate the importance of gene regulation and expression in determining many aspects of phenotype. More directly, these results demonstrate that differences in the pattern of gene expression can also lead to fundamental differences in how developmetal noise is propogated within individuals. This lends further support to the hypothesis of Leamy and Klingenberg [28] that the basis of developmental instability results from a stochastic component of developmental gene expression.

### Temperature and fluctuating asymmetry

Both environmental temperature and FA potentially interact with growth rate and size in ectothermic species and, therefore, they should indirectly influence each other. There are several ways that FA might respond to temperature. First, increased temperature may increase molecular perturbation, which may further act to increase overall levels of developmental noise during development. This should predictably increase FA with temperature. Second, increased temperature may act as a behavioral cue to shorten development time [41], thereby reducing the total time in which developmental errors may occur. This should predictably have the opposite effect, reducing FA as temperature increases. A third possibility is that a species specific optimal temperature exists. If so, FA should increase while approaching both the upper and lower thermal tolerance limits of organisms.

Only a few studies have directly investigated the relationship between FA and temperature. The results are conflicting. FA is found to increase on both sides of an "optimal" temperature [42-44], to be highest at low temperature [45], to simply increase with increasing temperature [46,47], or not to respond at all [48]. In none of these studies were genetic differences between individuals in populations controlled for, therefore, the relative contribution of internal developmental noise versus external environmental effect could not be assessed.

In this study, there is a clear overall decrease in FA with increasing temperature. This strongly supports that developmental instability is a function of developmental time rather than growth rate. Thus, the longer time spent in development and therefore, the more interrupted the growth process becomes, the larger the accumulation of multiplicative developmental errors. This was especially evident in the curve of centroid size-based FA, which closely follows the curve of development time. The slope of the temperature trend in mean FA is much steeper in dark morphotypes, which also have significantly higher FA, further indicating that environmental influences on FA are primarily related to individual developmental time. This result is consistent with the explanation of the basis of FA by Emlen et al. [24] and counters the assumption of Moller and Pomiankowski [9] that rapid growth is stressful and should, therefore, impart higher FA.

## Conclusion

This research indicates that within at least one morphological trait, the aphid wing, moderate levels of internal stochastic developmental noise do exist and potentially could impart some degree of insensitivity of FA to the environment. It perhaps also suggests that developmental accuracy may come at some significant cost that some organisms are unwilling to allocate towards certain aspects of their morphologies. Variation imposed by differences in this cost may account for the trait and species specificity of FA often observed among studies. Recently, it has been demonstrated that stochastic events occurring during the rapid growth phase of development can have profound effects on statistical qualities of fluctuating asymmetry, such as tail size and variance [30]. This also probably applies to other aspects of morphology, such as size and shape. Ultimately, it is the statistical properties of phenotypic distributions that combine with both selection pressure and interference by developmental instability to determine the movement of evolution on an adaptive landscape. In the early years of evolutionary theory, Fisher [49], Haldane [50], and Wright [51], who made the simplifying assumption of a direct relationship between genotype and phenotype, often dismissed the potential importance of development in the process of evolution. The resurgence of evo-devo has countered this traditional view with one where developmental processes and constraints hypothetically have played a primary role in determining what can evolve. However, until the actual relationship between genotype and phenotype is completely understood, including the role of developmental noise in obscuring this relationship, the truth that lies somewhere between these two extreme views will remain unresolved. In the future, investigation of relative levels of developmental noise across various species and species traits could help further illuminate one important aspect of the division between the information for building phenotype that ultimately lies in the genes and the actual individual phenotypes that are produced in natural populations.
